# Retraction: Resveratrol eliminates cancer stem cells of osteosarcoma by STAT3 pathway inhibition

**DOI:** 10.1371/journal.pone.0361240

**Published:** 2026-10-26

**Authors:** The PLOS One Editors

Following the publication of this article [1], concerns were raised about results presented in Figs 2-4, as well as the reporting of the study’s methodology. Specifically,

- There appear to be unusual and inconsistent repetitive features between the backgrounds of all Fig 2A panels.
- The Fig 3A MG-63 Vehicle panel of [1] appears more similar to the Fig 5A Dex+GYY panel of [2] than would be expected from independent results.
- The following panels appear to fully or partially overlap:
  - Fig 3D MG-63 Bcl-xL lane 1 and Fig 3D MNNG/HOS Cleaved caspase3 lane 2 when rotated and flipped.
  - Fig 3D MG-63 Cleaved caspase3 and Fig 3D MNNG/HOS Bcl-xL.
  - Fig 4A MG-63 p-JAK2 of [1] and Fig 4B HCT116 β-actin (42 kD) of [3, retracted in 4].
  - Fig 4A MG-63 p-STAT3 of [1] and Fig 4B flSREBP1(125 kD) of [3, retracted in 4].
  - Fig 4A MNNG/HOS p-STAT3 of [1] and Fig 4B mSREBP1 (65 kD) of [3, retracted in 4] when flipped.
- The article’s methodology reporting pertaining to animal research does not meet the journal’s Animal Research Reporting Guidelines policy in place at the time the manuscript was submitted.
- The Materials and methods section only reports the use of Student’s t-test, and does not explain how the analysis was adjusted for multivariate comparisons or corrected for multiple pairwise comparisons.

The authors did not respond to the journal’s communications pertaining to the above concerns. In the absence of author responses and the original data underlying the published results, these concerns cannot be resolved.

In light of the unresolved concerns, which call into question the reliability and integrity of the published results, the *PLOS One* Editors retract this article.

All authors either did not respond directly or could not be reached.
